# FLT3 in lineage specification and plasticity

**DOI:** 10.18632/oncotarget.499

**Published:** 2012-05-27

**Authors:** Sarah Greenblatt, Donald Small

**Affiliations:** ^1^ Department of Oncology, Johns Hopkins University School of Medicine, Baltimore, MD; ^2^ Department of Pediatrics, Johns Hopkins University School of Medicine, Baltimore, MD

**Keywords:** FLT3, B220, leukemia, lineage, differentiation

## Abstract

FLT3 is a receptor tyrosine kinase that is expressed in CD34^+^ hematopoietic stem/progenitor cells (HSPCs) and is important for both normal myeloid and lymphoid differentiation. FLT3 expression in Pax5 negative lymphoid precursors coincides with a window of multilineage differentiation potential in mice and humans. Recent work has shown that FLT3 activating mutations can collaborate with a Nup98-HoxD13 mutation to induce an aggressive acute leukemia. The leukemic initiating population in this model displayed properties of both lymphoid and myeloid precursors, making it a useful tool to study the role of FLT3 in lineage plasticity. Through a variety of assays, the leukemic initiating population was shown to be restricted to myeloid differentiation, suggesting that the B-lineage properties in these cells are due to the priming of lymphoid transcription programs in multipotent progenitors rather than a true capacity for B-cell maturation. The development of an undifferentiated myeloid leukemia in this model, also has implications for the role of FLT3 in the inhibition of myeloid differentiation. Here we discuss the insights gained from this model.

## INTRODUCTION

The commitment of cells to myeloid or lymphoid lineage development is considered to be an irreversible process, whereby distinct gene expression programs are triggered involving epigenetic changes in DNA and protein modification. However, there is evidence that multipotent progenitor cells prime several different lineage programs at low levels in order to respond rapidly to external stimuli [1]. Differentiation requires both an increase in lineage specific genes and a decrease in gene expression associated with alternative lineages. Therefore, dysregulation of gene expression by oncogenic mutations can lead to lineage conversion or multilineage properties in these primitive cells.

Activating mutations of the FLT3 receptor are some of the most common alterations in acute myeloid leukemia (AML) and have been extensively studied in patient samples, cell lines, and model systems [2-5]. We recently published a study describing a mouse model of cooperation between a knock-in of a FLT3 internal tandem duplication (ITD) mutation and a transgenic Nup98-HoxD13 (NHD13) translocation [6]. While the FLT3/ITD mutation alone induces a lethal myeloproliferative neoplasm (MPN) [7] and the Nup98-HoxD13 translocation alone produces a myelodysplastic syndrome (MDS), mice bred to express both mutations develop an acute leukemia with short latency and 100% penetrance.

Initially, the leukemia observed in this model appeared to express cell surface markers characteristic of both primitive myeloid and lymphoid development. The induction of a biphenotypic leukemia in this model would be surprising given the clinical data documenting the role of both Nup98 translocations and FLT3/ITD mutations in myeloid disease. The case reports describing patients harboring a Nup98-HoxD13 translocation have all reported myeloid malignancies; 1 case of therapy related acute erythroid leukemia and 3 cases with acute myelomonocytic leukemia [8-11]. To date, no Nup98 translocations have been observed in a patient with a B-cell malignancy [12]. Similarly, FLT3-ITD mutations are largely restricted to subtypes of AML, and are rarely reported in cases of acute lymphoblastic leukemia (ALL) [2, 13-14]. Finally, the FLT3/ITD-NHD13 model mimics the stepwise progression of a pre-leukemic disorder characterized by myelodyspasia, to overt leukemia that is frequently observed in patients. Approximately, 30% of MDS cases progress to AML, while the progression of MDS to ALL is rare [15].

### FLT3 in normal and malignant B-cells

Although FLT3 activating mutations appear to be primarily restricted to myeloid leukemias in patients, wildtype FLT3 is known to be expressed in lymphoid precursors and is expressed at low levels in nearly 100% of B-cell ALLs, suggesting a role for the receptor in early B-cell development [16-18]. Furthermore, subsets of ALL associated with specific chromosomal translocations such as MLL rearrangements have been shown to express FLT3 at high levels [19]. Although FLT3 activating mutations occur rarely in lymphoid leukemia, they occur at a higher frequency in subtypes of childhood leukemia such as hyperdiploid and MLL rearranged ALL [20-21].

In contrast to what is observed in patients, many mouse models that result in expression of FLT3 activating mutations through retroviral transduction of bone marrow or the generation of transgenic or “knock-in” mice have reported leukemias that express cell surface markers characteristic of biphenotypic or lymphoid development. For example, transduction of bone marrow with a FLT3 tyrosine kinase domain mutation results in a high proportion of lymphoid disease [22]. The combination of FLT3/ITD mutations with oncogenic fusions such as MLL-SEPT6 and AML1-ETO has also yielded a subset of mice with lymphoid disease [23-24]. In addition, a retroviral insertional mutagenesis screen in the FLT3/ITD knock-in background identified a high proportion of lymphoid leukemias [25].

### Regulation of FLT3 in murine B-cell development

One explanation for the development of a myeloid disease with early B-lymphoid properties in mice is that regulation of FLT3 expression is critical for early B-cell maturation. Gene knock-out studies have illustrated the importance of the balance between FLT3 and Pax5, a transcription factor that is critical for B-lineage commitment [26-27]. Pax5^−/−^ pre-B cells are susceptible to myeloid lineage switching when transduced with C/EBP α or the GATA family of transcription factors, but lose this ability after Pax5 induction [28]. This window of multilineage developmental potential is associated with FLT3 expression [29]. Holmes et al. have shown that Pax5 deficient proB cells express high levels of FLT3, but that it is repressed upon ectopic Pax5 expression. Further studies in mice have shown that knock-down or over expression of FLT3 can have a profound impact on B-cell development. FLT3^−/−^ mice have reduced numbers of B-cell precursors in the bone marrow, though normal numbers of functional B cells are present in the periphery and mice lacking FLT3 ligand have decreased numbers of myeloid and B-lymphoid progenitors [30-32]. Conversely, forced over expression of FLT3 or FLT3 ligand in mouse bone marrow results in decreased numbers of B220^+^CD19^+^ cells [29, 33]. In a more physiologically relevant system, where the FLT3/ITD mutation is expressed under the endogenous promoter, mice develop a block in differentiation at an early pro B-cell stage [34]. Taken together, these studies indicate that temporal control of FLT3 expression is critical for B-cell maturation.

Several murine models of hematopoietic malignancy have reported leukemias that co-express cell surface markers consistent with both myeloid and lymphoid cells, typically using B220 as evidence of B-cell differentiation. However, B220 appears early in hematopoietic differentiation and can also be expressed on many different cell types. Through sorting and transplantation of progenitor cell populations from a leukemic FLT3/ITD-NHD13 donor mouse, we showed that the ability to transplant the disease was restricted to a B220^+^ population with the immunophenotype of a multipotent progenitor. However, despite expression of B220, the leukemic initiating population was restricted to myeloid differentiation *in vitro*. This population had no other B-lymphoid properties. The cells did not display B-cell specific transcription factor expression or any other cell surface markers consistent with B-cell precursors. Although this population had D-to-J rearrangements of the IgH locus, they did not show any signs of V-to-DJ rearrangements, which are definitive for B- lymphoid cells. Single cell analysis of CD19^−^ B-precursor populations in the mouse bone marrow have shown that recombination activating genes can be detected at low levels in most multipotent progenitor cells, a population that retains the capacity for myeloid differentiation. Thus, the presence of D-to-J rearrangements and surface B220 expression may be due to the priming of lymphoid differentiation programs at low levels in multipotent progenitors, rather than evidence for lymphoid differentiation.

### FLT3 and B220 expression

There is previous evidence of a connection between FLT3 signaling and the expansion of hematopoietic stem and progenitor cells (HSPCs) expressing B220. One study identified a population of CD19^−^B220^+^c-KIT^+^FLT3^+^ cells as early B-lymphoid precursors in juvenile mouse bone marrow that is highly dependent on FLT3 expression for survival [35-36]. These cells possess the potential to produce both lymphoid and myeloid colonies *in vitro*. Other studies observed that FLT3 ligand induces the outgrowth of Mac-1^+^/B220^+^ mouse bone marrow progenitor cells restricted to macrophage differentiation that co-express early B-cell associated genes [37]. Pre-B-cell progenitors with myeloid characteristics have also been described when murine cells are transformed with several oncogenic receptor tyrosine kinases [38]. Together, these studies suggest that a population of pre-B cells with bilineage potential exists within juvenile mice that is dependent on FLT3 expression and highly susceptible to transformation.

Recent work has shown that a leukemia initiating population with lymphoid characteristics can exist in patients with AML, suggesting that the presense of a myeloid restricted B220 expressing cell is not a mouse specific phenomenon [39]. In this study, CALM/AF10 positive AML samples were shown to co-express B220 and CD34, a marker used to define HSPC populations, and had detectable clonal immunoglobulin rearrangements. Rearrangements of the IgH locus have been observed in a significant number of AML cases, suggesting that a larger subset of leukemias may also be derived from a progenitor population with lymphoid characteristics [40].

### FLT3 in lineage specification

The presence of a FLT3 activating mutation can also affect the lineage restriction of HSPCs. Although FLT3 mutations are typically categorized by their ability to promote survival and proliferation, accumulating data has suggested that FLT3/ITD activating mutations can also contribute to a block in differentiation [41-42]. Radomska et al. has shown that constitutive activation of FLT3 can inhibit CEBPα function through ERK1/2 mediated phosphorylation, resulting in a block in differentiation in both cell lines and patient samples. Treatment of primary blasts with the FLT3 inhibitor CEP701 was able to partially reverse this differentiation block, as evidenced by increased expression of CEPBα and Pu.1 and enhanced granulocytic differentiation. This work suggests that signaling downstream of the constitutively activated receptor may alter the activity of transcription factors that are critical for myeloid differentiation.

The distribution of disease in the FLT3/ITD-NHD13 model also supports the role of FLT3 mutations in the impairment of differentiation. Although the NHD13 alone mice all develop a myelodysplastic syndrome, a subset of these mice are known to progress to differentiated leukemias of both the myeloid and lymphoid lineages [43]. These mice develop a variety of differentiated myeloid neoplasms including acute megakaryocytic, erythroid, and myelomonocytic leukemias. However, when this mutation is combined with the FLT3/ITD knock-in mutation, mice develop strictly minimally differentiated myeloid leukemias. This difference in leukemia subtype, suggests that the presence of a FLT3/ITD mutation in this system results in the transformation of very early myeloid precursors that are impaired in maturation. Accordingly, HSPCs isolated from the FLT3/ITD-NHD13 mice showed an impaired ability of to differentiate in vitro [Greenblatt and Small, unpublished data], even compared to the NHD13 or FLT3/ITD alone mice [44].

In the FLT3/ITD-NHD13 mice, an early genetic event leading to loss of the wildtype FLT3 allele allows us to track cells originating from the leukemic initiating population. Loss of heterozygosity is never found in megakaryocytic/erythroid progenitors or CD19^+^ B-cells populations, indicating that the leukemic initiating population does not contribute to these lineages. These data provide further evidence that FLT3/ITD mutations play a role in the regulation of differentiation and lineage restriction of the leukemic stem cell. The contribution of both mutations to impairment in differentiation may also explain why the concomitant expression of a Nup98 translocation and FLT3/ITD mutation results in a short latency to disease in mice and a poor prognosis in patients.

## CONCLUSION

This cumulative work suggests that regulation of FLT3 expression is critical for both myeloid and lymphoid differentiation. Disturbance of FLT3 through over-expression or constitutive activation by FLT3 activating mutations can result in leukemias with promiscuous lineage properties. While these properties may make the cells more difficult to treat, they may also provide a unique target for treatment since the leukemic initiating population may express a cell surface profile distinct from normal stem cells.
